# Cellular and Molecular Profiling of Tumor Microenvironment and Early-Stage Lung Cancer

**DOI:** 10.3390/ijms23105346

**Published:** 2022-05-11

**Authors:** Radu Pirlog, Paul Chiroi, Ioana Rusu, Ancuta Maria Jurj, Liviuta Budisan, Cecilia Pop-Bica, Cornelia Braicu, Doinita Crisan, Jean-Christophe Sabourin, Ioana Berindan-Neagoe

**Affiliations:** 1 Research Center for Functional Genomics, Biomedicine and Translational Medicine, “Iuliu Hatieganu” University of Medicine and Pharmacy, 400337 Cluj-Napoca, Romania; pirlog.radu@umfcluj.ro (R.P.); chiroipaul@gmail.com (P.C.); anca.jurj@umfcluj.ro (A.M.J.); liviuta.budisan@umfcluj.ro (L.B.); cecilia.bica8@gmail.com (C.P.-B.); corneliabraicu@yahoo.com (C.B.); 2 Department of Pathology, Regional Institute of Gastroenterology and Hepatology, “Iuliu Hațieganu” University of Medicine and Pharmacy, 400186 Cluj-Napoca, Romania; ioana.russu@gmail.com; 3 Department of Morphological Sciences, “Iuliu Hațieganu” University of Medicine and Pharmacy, 6 Pasteur Street, 400349 Cluj-Napoca, Romania; 4 Pathology Department and INSERM U1245, Rouen University Hospital, Normandy University, 76000 Rouen, France; jean-christophe.sabourin@chu-rouen.fr

**Keywords:** early-stage lung cancer, tumor microenvironment, p53, E-cadherin, CD4, CD8, hsa-miR-25-3p, hsa-miR-29b-3p, hsa-miR-181a-5p, hsa-miR-205-5p

## Abstract

Lung cancers are broadly divided into two categories: non-small-cell lung carcinoma (NSCLC), which accounts for 80–85% of all cancer cases, and small-cell lung carcinoma (SCLC), which covers the remaining 10–15%. Recent advances in cancer biology and genomics research have allowed an in-depth characterization of lung cancers that have revealed new therapy targets (*EGFR*, *ALK*, *ROS*, and *KRAS* mutations) and have the potential of revealing even more biomarkers for diagnostic, prognostic, and targeted therapies. A new source of biomarkers is represented by non-coding RNAs, especially microRNAs (miRNAs). MiRNAs are short non-coding RNA sequences that have essential regulatory roles in multiple cancers. Therefore, we aim to investigate the tumor microenvironment (TME) and miRNA tumor profile in a subset of 51 early-stage lung cancer samples (T1 and T2) to better understand early tumor and TME organization and molecular dysregulation. We analyzed the immunohistochemistry expression of CD4 and CD8 as markers of the main TME immune populations, E-cadherin to evaluate early-stage epithelial-to-mesenchymal transition (EMT), and p53, the main altered tumor suppressor gene in lung cancer. Starting from these 4 markers, we identified and validated 4 miRNAs that target *TP53* and regulate EMT that can be further investigated as potential early-stage lung cancer biomarkers.

## 1. Introduction

Lung cancer is the leading cause of cancer-related death worldwide, accounting for more than 1.8 million fatalities each year. It is also the second most frequent type of malignancy, with more than 2.2 million cases diagnosed annually [1]. Lung cancers are broadly divided into two main histological groups: NSCLC, which accounts for 80–85% of all lung cancer cases, and SCLC, consisting of 10–15%. NSCLC can be further divided into three principal histological subtypes: lung adenocarcinomas (LUADs) (45–60% of cases), squamous cell carcinoma (LUSC) (20–25% of cases), and neuroendocrine carcinomas (NE LC) (10–15%) [1,2]. These histological types differ in terms of treatment approaches and overall survival (OS). NSCLC is the most studied type, with multiple targeted therapies available and with a 5-year OS rate of 23%, while SCLC is treated mainly with platinum-based chemotherapy and has a median survival time of less than 1 year [3,4]. This high mortality is due to late diagnosis and the paucity of effective screening regimens. Lung cancer detected in early-stage has a 5-year OS of up to 70% [5], highlighting the need for better early-stage biomarkers.

Chest X-ray, computed tomography, magnetic resonance imaging, positron emission tomography, sputum analysis, and lung biopsy are all common clinical investigations currently used for identifying lung cancer. However, despite recent advances in cancer screening and diagnosis, 57% of all patients are detected only after the tumor has progressed to the metastatic stage. Under these circumstances, the remission chances are minimal, and the 5-year survival rate for metastatic lung cancer is less than 6% [6]. Therefore, identifying new methods for early-stage lung cancer detection is a central focus of cancer research. In this regard, understanding the alterations in the lung tumor microenvironment (TME) throughout the early stages of cancer evolution could be a viable avenue for biomarker discovery [7,8].

The main risk factor associated with lung cancer is tobacco smoking, which is responsible for about 80–90% of all lung cancer cases. It triggers an aggressive mutational burden, an increase in cytosine to adenine nucleotide transversions, and an enhancement in *KRAS* and *TP53* mutations. *KRAS* mutations are the most common driver mutations, being present in about 35% of NSCLC cases [6]. *TP53* is the most important tumor suppressor gene and is the target of multiple mutations early in lung cancer tumorigenesis, its alterations being present in more than 90% of SCLCs and 50% of NSCLCs [9,10,11]. 

Recent advances in cancer biology and genomics research have allowed us to characterize the mutational landscape of lung cancer, and, in recent years, next-generation sequencing (NGS) has become an essential tool for diagnosis and effective therapeutic management [9,10]. Improvements in the understanding of lung cancer genomics have led to the identification of major driver mutations genes, including *EGFR*, *KRAS*, *ALK*, *BRAF*, *HER2*, *PIK3CA*, *AKT1*, *MAP2K1*, and *MET* [12,13]. The identification of these mutations has led to major progress in therapy for LUAD patients, with the introduction of tyrosine kinase inhibitors and, more recently, KRAS G12C inhibitors [14,15,16]. The identification of these mutations changed clinical practice and pathological diagnosis. Nowadays, searching for driver mutations is mandatory for a complete and accurate diagnosis. 

From the early development of the lung cancer cellular niche, the tumor starts to organize a complex cellular and molecular habitat that is known as the TME. This entity consists of a complex stromal and cellular network of tumor cells, various normal and immune cell populations, and molecules trafficked in this environment [17]. The principal non-tumoral populations are represented by tumor-infiltrating lymphocytes (TILs), tumor-associated fibroblasts, tumor-associated macrophages, and endothelial progenitor cells [18]. This ecosystem is designed to support the tumor from its early development in acquiring the hallmarks of cancer and support its progression toward more advanced stages [19]. The interest in the study of TME increased with the introduction of anti-PD1/anti-PD-L1 and anti-CTLA4 immunotherapy. The introduction of immunotherapy managed to reactivate the dormant immune population from the environment and became a valuable resource in lung cancer therapy [20]. Currently, all major cellular components of the TME are being intensively studied to identify new targets that can enhance their antitumor activity [21].

Epithelial-to-mesenchymal transition (EMT) is a dynamic process that takes place early in lung cancer tumorigenesis, being an essential step for tumor cells to acquire invasive and metastatic potential [22,23]. The EMT phenotype was identified as early as stage IA in a cohort of LUAD patients. The presence of the mesenchymal phenotype, reduced E-cadherin and high Vimentin expression, was associated with shorter disease-free survival and reduced OS [24]. Additionally, circulating tumor cells, a marker of EMT, were shown to be detected as early as in situ stages of lung cancer in the blood of patients, supporting the early onset of EMT [25]. A better understanding of EMT in the early stages of cancer is essential for understanding tumor progression and for identifying the molecular mechanisms that can be modulated to limit progression and spread to distant sites [26]. 

NGS, including single-cell analysis, has been used in TME investigation [18,21,27,28]. These methods have shown promising results, highlighting the possible prognostic role of tumor-infiltrating lymphocytes (TILs) in lung cancer. Additionally, approaches that used immunohistochemistry (IHC) for specific TIL subpopulations revealed that the abundance of CD3, CD4, CD8, and FOXP3+ subpopulations can be used as an independent prognostic marker for patient outcome [18,29]. 

Another conceivable source of biomarkers is represented by the non-coding RNAs (ncRNAs), especially microRNAs (miRNAs); they are short (18–28 nucleotides long) ncRNA sequences found to be involved in the post-transcriptional regulation of gene expression. Thus, miRNAs are potent regulators of cell proliferation, differentiation, development, and apoptosis, among other cellular processes. Furthermore, these ncRNAs were also found involved in various malignancies, including lung cancer [30,31,32]. Moreover, under certain circumstances, miRNAs can act as oncogenes or tumor suppressors. Therefore, different cancer hallmarks, such as sustaining proliferative signals, evading growth suppressors, resisting cell death, activating invasion and metastasis, and initiating angiogenesis, have been linked to dysregulated miRNAs. Hence, a growing body of studies has pinpointed the possible value of miRNAs as potential biomarkers for cancer diagnosis, prognosis, and therapeutic targets. However, additional investigations and validations are required to further unlock their use in clinical practice [33,34,35]. 

### Study Design

Based on bioinformatics analysis using dysregulated genes in early-stage lung cancer, TME analysis, and literature search, we were able to identify 4 miRNAs (hsa-miR-29b-3p, hsa-miR-181a-5p, hsa-miR-25-3p, and hsa-miR-205-5p) that target the *TP53* gene, modulate EMT pathway in lung cancer and have the potential of being used as a diagnostic panel for early-stage lung cancer. 

We investigate a 4-miRNA panel for early-stage lung cancer diagnosis, consisting of two tumor suppressors and two tumor promoter miRNAs known for their roles in essential cancer regulatory processes, such as EMT, angiogenesis, metastasis, and clinical parameters such as response to therapy and OS. 

In this study, we present a translational approach for biomarker identification in early-stage lung cancer. We used a comprehensive characterization of the selected cases that included morphology, IHC, bioinformatics analysis, and investigation of specific miRNAs expression using qRT-PCR. We included early-stage LUAD, LUSC, and NE LC to better understand lung cancer progression and TME organization in the early stages of carcinogenesis across the different histologic subtypes. 

## 2. Results

### 2.1. Patients’ Characteristics

The mean age in our group was 61 years, ranging from 32 to 82 years old. The sex distribution included 23.5% (12/51) females and 76.5% (39/51) males. The 5-year survival was 68.6% (35/51) in our study group. No statistically significant difference in survival was seen among the three main histology groups. 

### 2.2. Morphologic Characteristics

Tumor histology, staging, degree of differentiation, and morphological characteristics are presented in Table 1. Our cohort included 22 LUADs (43.1%), 21 LUSCs (41.1%), and 8 NE LCs (15.8%). 

The pathological stages correspond to early-stage lung cancer, with 47% of stage IA, 37.2% stage IB, and 15.7% of stage IIA cases. The degree of differentiation is moderately differentiated in 37.2% of cases and poorly differentiated in 41.1% of cases. There was a statistically significant difference between tumor histology and differentiation grade, with a NE LC showing the highest rate of well-differentiated cases (62.5%; 5/8). 

**Figure 1:**
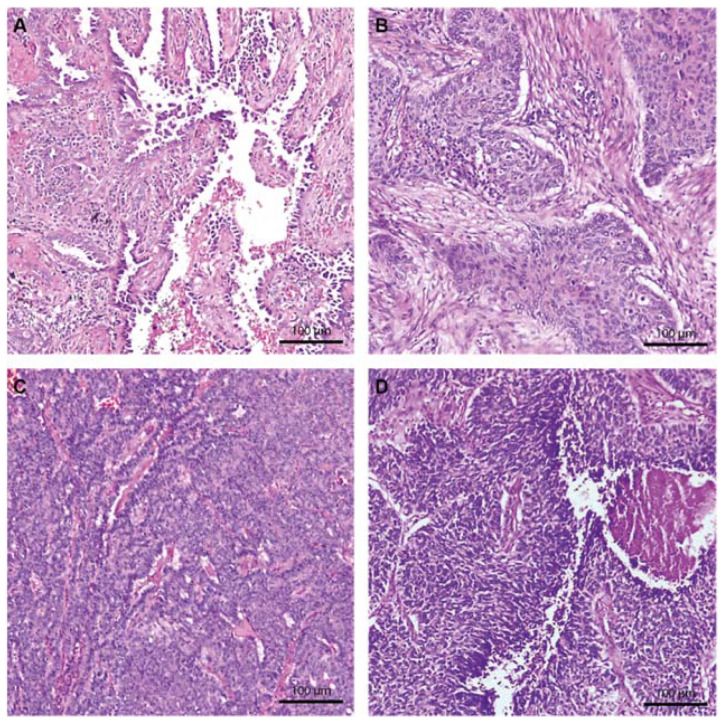
Hematoxylin and eosin staining of the principal lung cancer subtypes at 100×. (**A**) LUAD shows the typical glandular architecture and atypical large nuclei. (**B**) LUSC with solid tumor islands and a fibrotic stroma. (**C**) NE LC with a dense cellular proliferation. (**D**) NE LC with intratumor necrosis, atypical nuclei, and nuclear molding aspects, suggestive of SCLC.

Intratumor necrosis was present in 76.4% of the cases and atypical nuclei in 41.1% of the cases (Figure 1). LUSC showed a higher rate of intratumor necrosis, with all cases in our cohort showing areas of tumor necrosis (*p* = 0.003). Nuclear atypia was statistically significantly different among the three tumor types, with LUSC cases having the highest rate of nuclear atypia in 61.9% (13/21) of the cases and NE LC the lowest in 12.5% (1/8) of the cases (Table 1). 

### 2.3. Immunohistochemistry

**Figure 2:**
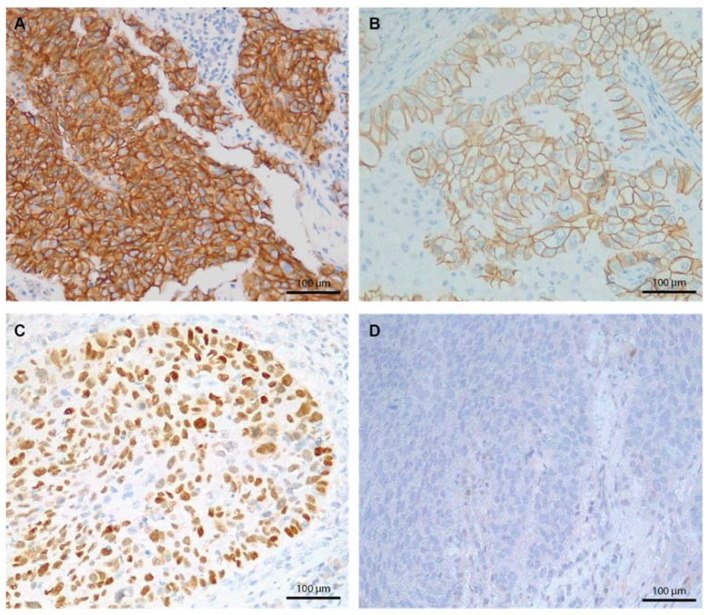
Immunohistochemistry staining 100×. (**A**) Lung neuroendocrine tumor showing an E-cadherin positive continuous membranous staining, score 3+. (**B**) LUAD E-cadherin positive membranous staining, score 2+. (**C**) LUSC, p53 positive nuclear staining, score 1. (**D**) LUSC, p53 negative staining, score 0.

Our cases were analyzed using IHC staining for E-cadherin and p53. E-cadherin IHC was intensely positive in 37.3% of the cases (19/51) and moderately positive in 67.7% of the cases (32/51). P53 IHC was positive in 67.7% (32/51) of the cases (Figure 2). P53 positivity was higher in LUAD, where 76.2% (16/21) of the cases were positive. No statistically significant differences regarding the E-cadherin and p53 IHC were seen among the three histology groups. Detailed IHC profiles according to the histologic subtype are presented in Table 1.

### 2.4. Tumor Microenvironment

**Figure 3:**
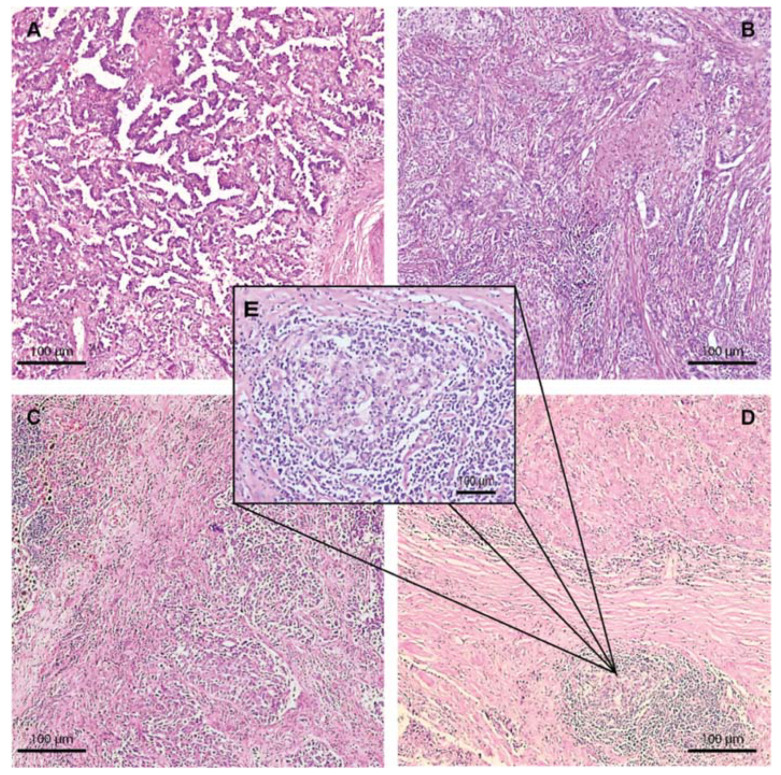
Hematoxylin and eosin staining of LUADs showing the different grades of peritumoral and intratumor immune infiltrate, 50×. (**A**) LUAD showing peritumoral and intratumor immune infiltrate score 1. (**B**) LUAD with a medium density of the immune infiltrate, score 2. (**C**) LUAD with a high abundance of immune infiltration in both peritumoral and intertumoral compartments. (**D**) LUAD showing tertiary lymphoid structures situated at the periphery of the tumor located in a fibrous stroma. (**E**) High magnification at 200× of the tertiary lymphoid structure with the active germinal center.

TME analysis showed a moderate-to-high peritumoral inflammatory infiltrate in 80.4% of the cases, whereas intratumor lymphocytes were moderate in 15.6% and low in 80.4% of the cases (Figure 3). TILs were more abundant at both stromal and intratumor compartments in LUAD and LUSC cases. In NE LC, 87.5% (7/8) of the cases showed a low abundance of TILs. 

The TLS were present in 74.5% of the cases and showed active germinative centers in 34.2% of TLS (Table 1) (Figure 3). LUADs showed a higher abundance of active germinative centers (47.1%; 8/17) compared with LUSC (23.5%; 4/17) and NE LC (25%; 1/4). No statistically significant differences among histology groups were observed. 

**Figure 4:**
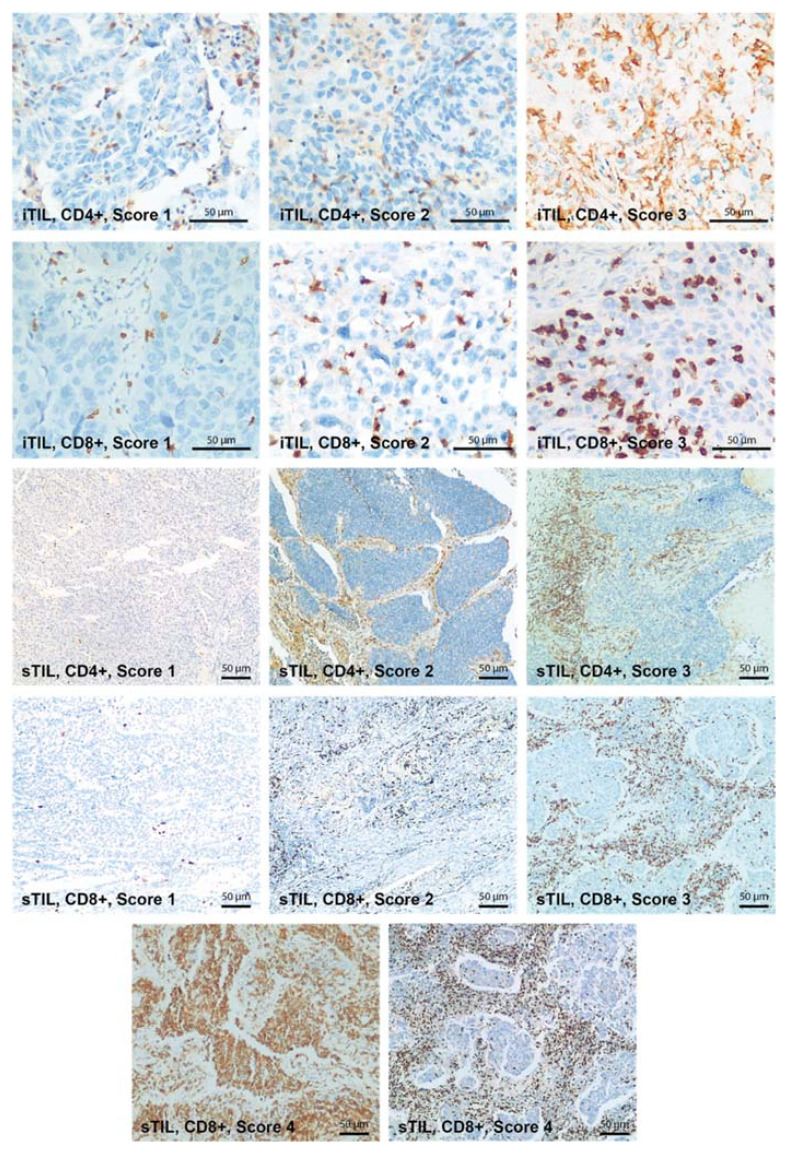
IHC staining for CD4 and CD8 cells shows the difference in abundance of the inflammatory infiltrate in the peritumor and intratumor compartments. The images of the intratumor compartment are at 200×, and the peritumor compartment images are at 100× magnification. iTIL—intratumor tumor-infiltrating lymphocytes. sTIL—stromal tumor-infiltrating lymphocytes.

IHC analysis was used to characterize the TILs populations from the stromal and intratumor compartments (Figure 4). The stromal compartment cellular composition consisted mainly of CD4 lymphocytes that were present in high and very high abundance in 84.4% of the cases. Statistically significant differences were observed among the three histologic groups, with NE LC showing the lowest abundance of stromal CD4 TILs and a low abundance in 50% of the cases. 

The CD8 cells were lower in abundance compared with CD4 cells, showing a moderate and low presence in 64.7% of the cases. The intratumor compartment was scarce in TILs, with minor differences between CD4 and CD8 TILs, but with a slight predominance in CD8 TILs (Table 1). 

### 2.5. Bioinformatics Pipeline and In Silico Analysis

**Figure 5:**
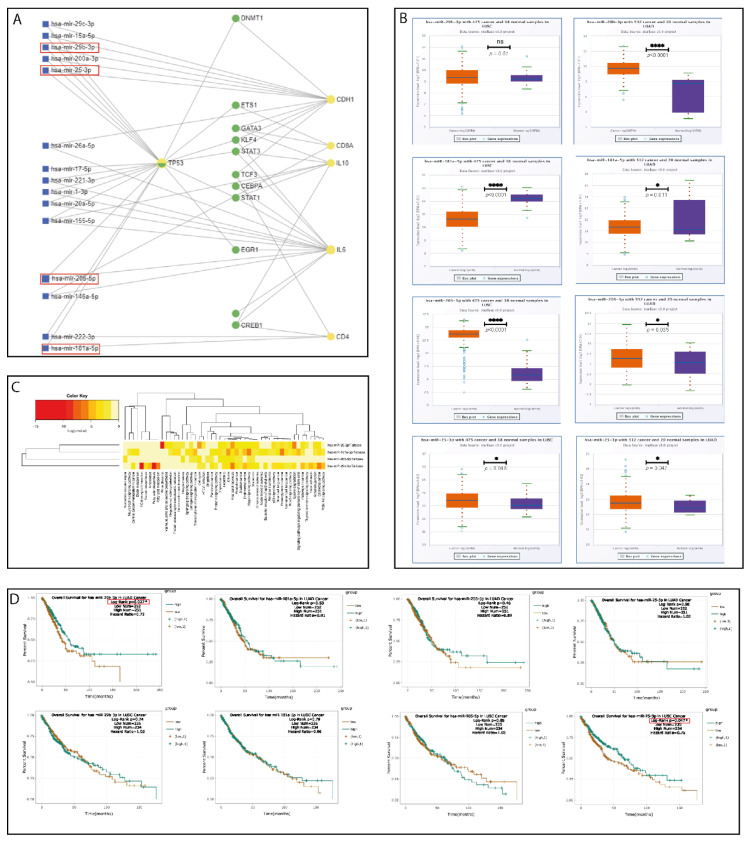
(**A**) Bioinformatics analysis showing the interaction network between the genes of interest and specific miRNAs. Red boxes highlight miRNAs selected for further validation. (**B**) TCGA database expression analysis of the selected miRNAs in LUADs and LUSCs. (**C**) Heatmap showing the principal metabolic pathways and cancers in which the 4 selected miRNAs are involved. (**D**) Survival analysis based on the TCGA database on LUAD and LUSC cases for the selected miRNAs. The red box highlights the positive impact on survival of upregulation of the hsa-miR-29b-3p in LUAD patients and hsa-miR-25-3p in LUSC patients. ns—not significant; * *p* ≤ 0.05; **** *p* ≤ 0.0001.

Based on the selected tumor (E-cadherin and p53) and TME IHC biomarkers (CD4 and CD8), we used the miRNET software to generate a network of interactions between miRNAs and their target genes. *TP53* emerged as the network’s main hub gene, being centrally located and connected with multiple miRNAs, followed by the *E-cadherin* gene (*CDH1*), *IL-6,* and *CD4*. Based on the network analysis and literature search for miRNAs involved in EMT regulation in lung cancer, 4 miRNAs were selected for additional investigation (Figure 5A—red square) (Table 2).

These 4 miRNAs were selected for their potential role as a diagnostic panel for early-stage lung cancer. Among the 4 miRNAs, hsa-miR-181a-5p was extensively reviewed by our team for its role in lung cancer and showed an association with the OS [41,42]. Hsa-miR_181a-5p was detected in the serum of lung cancer patients and proposed as a non-invasive biomarker [46].

Hsa-miR-205-5p is a tumor promoter miRNA that has been investigated by multiple studies for its role in the diagnosis of lung cancer; having been detected both in tumor tissue and plasma, it is generally overexpressed in NSCLCs. Interestingly, although it was associated by multiple studies with lung cancer progression and metastasis, following a meta-analysis, its upregulation was associated with an improved prognosis [47,48,49]. Hsa-miR-205-5p was also investigated by our team in colorectal cancer and showed its regulator role on the EMT pathway by modulation of *ZEB1* and E-cadherin expression [44]. These two miRNAs, hsa-miR-181a-5p and hsa-miR-205-5p, were selected based on our experience and translational potential for early-stage lung cancer diagnosis.

Hsa-miR-25-3p is a tumor promoter miRNA valuable for the diagnosis of lung cancer. Its upregulation in serum samples was observed across multiple studies on the Chinese population on NSCLC tumor samples [50]. On the other hand, in a study performed on an Indian cohort hsa-miR-25-3p was found to be downregulated in the serum of NSCLC patients [51]. Therefore, the expression of this miRNA in lung cancer is under investigation, being of interest to understand its expression dynamics in early and advanced cancer stages. 

Hsa-miR-29b-3p is a tumor suppressor miRNA in the context of lung cancer, where it was found to inhibit proliferation, migration, and invasion [40]. Hsa-miR-29b-3p is generally downregulated in advanced NSCLC; its inhibition is associated with EMT, chemotherapy, and radiotherapy resistance and poor overall survival [38,39,52]. Still, there is very little evidence available regarding the role of hsa-miR-29b-3p in the early stages of lung cancer development [53] and the dynamics of this miRNA. 

The differential expression of these 4 miRNAs was analyzed using the LUAD and LUSC cases from the TCGA database (Figure 5B). The limitation of TCGA database analysis is the lack of neuroendocrine lung cancer samples. A heatmap was generated to identify the expression level of these miRNAs in essential cancer tissues and their involvement in cancer metabolic processes (Figure 5C). The impact of these 4 miRNAs’ expression on patient survival showed that high hsa-miR-29b-3p is associated with an increase in OS in LUAD cases (*p* = 0.027) and upregulation of hsa-miR-25-3p is associated with a better OS in LUSC cases (*p* = 0.047) (Figure 5D). 

### 2.6. Validation of the Selected miRNA Panel on FFPE Tumor Tissue

**Figure 6:**
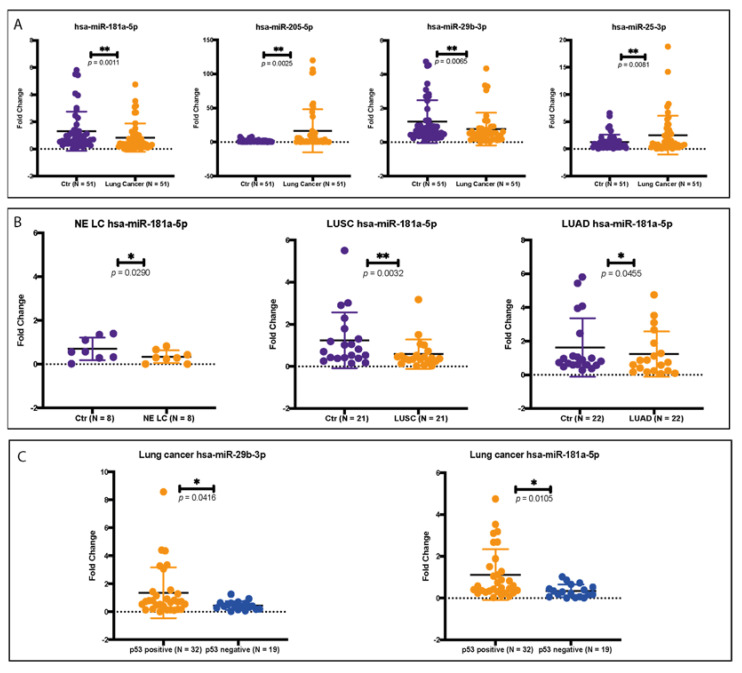
(**A**) qRT-PCR analysis of the selected miRNAs on our 51 samples of early-stage lung cancer. For each miRNA, we analyzed the difference in expression level between the tumor and adjacent normal tissue. The *p*-value is indicated for each miRNA with its corresponding significance. (**B**) The differences in hsa-miR-181a-5p expression in all three main histologic subtypes. Hsa-miR-181a-5p was found to be significantly downregulated in each of the cancer subtypes. (**C**) Hsa-miR-29b-3p and hsa-miR-181a-5p expression in early-stage lung cancer according to p53 IHC staining. The two miRNAs are upregulated in p53 IHC-positive tumors. LUSC—lung squamous cell carcinoma. LUAD—lung adenocarcinoma. NE LC—neuroendocrine lung cancer. * *p* ≤ 0.05; ** *p* ≤ 0.01.

The expression level of selected miRNAs was analyzed on RNA extracted from FFPE tumor tissue using qRT-PCR. We compared the differential expression of the selected miRNAs between adjacent normal tissue and tumor tissue in our 51 cases of early-stage lung cancer. The differential expression analysis revealed a statistically significant downregulation of hsa-miR-29b-3p and hsa-miR-181a-5p in tumor tissue. Hsa-miR-205-5p and hsa-miR-25-3p expression was upregulated in lung cancer tumor tissue when compared with adjacent normal tissue controls (Figure 6A).

When analyzing the differential expression according to histology type for hsa-miR-29b-3p, hsa-miR-205-5p, and hsa-miR-25-3p, the expression patterns were maintained but without achieving statistical significance. The expression level of hsa-miR-181a-5p was significantly downregulated in tumor tissue across all three main histological subtypes (Figure 6B).

MiRNAs expression was assessed according to tumor p53 and E-cadherin IHC expression. Hsa-miR-29b-3p and hsa-miR-181a-5p were significantly upregulated in p53 IHC-positive early-stage lung cancers when compared with p53-negative tumors (Figure 6C). No statistically significant difference was observed for hsa-miR-25-3p and hsa-miR-205-5p. Differences in E-cadherin IHC expression were not associated with miRNA expression dysregulation.

## 3. Discussion

We performed a translational analysis of the tumor and TME in early-stage lung cancer with the aim of identifying the main patterns of TME organization and tumor molecular alterations. The IHC profile of E-cadherin and p53 markers allowed us to evaluate early-phase EMT and the loss of the main tumor suppressor gene. E-cadherin is a glycoprotein that plays an important morphogenetic role in epithelial cell stabilization by maintaining intercellular connections through calcium-dependent adhesion. Additionally, it regulates cancer cell differentiation and reduces cancer cells’ ability to spread beyond their local site. Thus, reduced or absent E-cadherin expression in several cancers is linked to impaired differentiation and enhanced metastatic capacity. Depletion of E-cadherin expression causes EMT, which enhances the metastatic potential. Deconstruction of cell polarity, cytoskeleton restructuration, and changes in signaling pathways all contribute to EMT, which increases motility and promotes metastasis by increasing cancer cell invasiveness, resulting in a poor prognosis [54]. EMT signature has also been inversely associated with T-cell infiltration in NSCLC [55,56]. In our cohort, the E-cadherin staining was present in all the investigated cases, but a difference in intensity was noted among samples, with 37.3% of cases having high-intensity staining and 62.7% of these samples expressing a moderate staining intensity. This loss of staining intensity can be interpreted as an early sign of EMT, as it was shown in recent years that EMT in lung cancer is a process that starts from the early stages [24]. 

P53 is a tumor suppressor protein that controls cell division and proliferation that has also been linked to early lung cancer carcinogenesis [57,58]. The presence of p53 IHC staining generally indicates a mutation in the *TP53* gene [59]. In our series, p53 IHC was positive in 62.7% of the cases, supporting the importance of p53 mutation as an early-mutational event in early-stage lung cancer. Higher positivity rates were found in groups that included more advanced stages of lung cancer [60].

A critical element in lung cancer initiation and progression is represented by the organization of the TME, which takes place early in tumorigenesis [8,54,61]. Consequently, decoding the lung cancer-associated TME heterogeneity into a collection of prognostic, diagnostic, or predictive biomarkers has become an area of intensive research. Since then, several valuable findings have pinpointed the TME as a potential source for novel early-stage lung cancer biomarkers [8,58,62]. An important constituent at this level is represented by the immune cell populations, which are involved in the immune surveillance and tumor immune escape mechanisms. These two processes are in a dynamic equilibrium, with immune surveillance slowing tumor progression through tumor cell identification and suppression and progressing tumor immune escape by reducing the antitumor activity of the immune compartment as the tumor starts to produce inhibitory molecules and secrete cytokines [63,64]. Moreover, the TME-related immune signature has led to further investigations, especially in LUAD cases, where its diagnostic and prognostic role has been recently proposed [65]. In this regard, TILs play a significant role in the tumor immune milieu and can be a reliable early-stage biomarker [29,66,67]. Two of the most studied TILs that are believed to be associated with the early stages of lung cancer are CD4 and CD8 [68,69,70], commonly known for their important roles in the regulation of both antitumor and protumorigenic processes [71,72]. Using the IHC characterization of TILs, we found that CD4 lymphocytes were high and very high within the stromal compartment in 84.4% of the early-stage lung cancer cohort, while the CD8 cells were lower in abundance, showing a moderate and low abundance in 64.7% of the cases. Meanwhile, the intratumor compartment was scarce in TILs, with minor differences between CD4 and CD8 TILs. Therefore, our findings take us a step closer to validating the association between TILs (CD4/CD8) and early-stage tumorigenesis in lung cancer [18,70]. The presence of CD4 cells was correlated with hsa-miR-181a expression, as indicated by the in silico analysis Figure 5A and further validated on the TCGA dataset and our tumor samples. The tumor suppressor hsa-miR-181a was upregulated in the adjacent normal tumoral tissue, which contains the cellular elements of the TME, and downregulated at the tumoral level. 

The immune cell populations in the TME were shown to be organized in cellular aggregates that resemble secondary lymphoid organs in morphology and their composition being commonly defined as TLS. Moreover, histologically, these structures were shown to become phenotypically active when they start to develop germinal centers [73]. These structures are partially involved in mediating the host immune response, becoming a subject of interest in the study of TME [74,75]. In early-stage lung cancer, TLS were shown to actively drive the immune response against tumor cells, which is generally considered a sign of active tumor immunity and positive prognosis [70,76,77,78]. In our group, TLS were present in 74.5% of the early-stage lung cancer cases, and among these, 34.2% of cases with TLS showed the presence of active germinal centers (Figure 3). LUADs showed a higher presence of active germinal centers, suggesting they are more immunologically active compared with LUSCs and NE LCs. 

In the past decade, our understanding of cancer biology has quantitatively and qualitatively increased, due to the intensive genomic studies, especially regarding the roles of miRNAs in human cancers [79,80]. Different cancers have different miRNA expression levels, distinctive from those within normal tissues. Thus, abnormal miRNA expression patterns have been oftentimes considered a crucial carcinogenesis marker and may have the potential of becoming novel biomarkers for minimally invasive, early-stage lung diagnosis [53,71].

In our study, we performed a bioinformatics analysis starting from the tumor and TME markers, and, based on a literature search, we refined the analysis and selected 4 miRNAs, hsa-miR-29b-3p [81,82], hsa-miR-181a-5p [46,83], hsa-miR-25-3p [50,84,85], and hsa-miR-205-5p [49,82,86], that regulate EMT and have the potential of being used as diagnostic biomarkers in lung cancer. 

Based on TCGA database analysis, the tumor suppressor miRNA hsa-miR-181a-5p was downregulated in both LUAD and LUSC tumors, thus losing its important role in limiting cancer development. Hsa-miR-29b-3p was upregulated in LUAD cases without any difference in LUSC cases. The other two investigated miRNAs, hsa-miR-25-3p and hsa-miR-205-5p, had higher expression levels in both types, LUSC and LUAD, when compared with controls, thus confirming their oncogenic modulatory roles. The hsa-miR-29b-3p miRNA showed a positive effect of upregulation for LUAD patients’ OS, and hsa-miR-25-3p showed a similar effect in LUSC cases. 

Our expression analysis of the 4 miRNAs in our cohort of early-stage lung cancer showed a statistically significant downregulation of hsa-miR-29b-3p and hsa-miR-181a-5p, while hsa-miR-205-5p and hsa-miR-25-3p expression were upregulated in lung cancer tumor tissue when compared with the adjacent controls. 

The 4-miRNA panel consists of two tumor suppressors and two tumor promoter miRNAs. The investigation of this miRNA panel allows the evaluation of internal tumor physiology, indicating the change towards a more invasive phenotype by downregulation of tumor suppressor miRNA, upregulation of oncomiRs, inhibition of tumor suppressor genes, such as *TP53,* and activation of EMT. In our cohort, hsa-miR-29b-3p, a known tumor suppressor miRNA that is generally downregulated in advanced lung cancer, was upregulated early-stage lung cancer samples. High levels of hsa-miR-29b-3p are associated with radiosensitivity and chemosensitivity and good OS. Hsa-miR-29b-3p upregulation in early stages is in accordance with the positive outcomes of these tumors and can be used as a predictive biomarker [38,39].

Therefore, this 4-miRNA panel should be further validated in independent cohorts for its diagnostic role. As all of the miRNAs are involved in the regulation of EMT, it would be of interest to assess the expression dynamics of this miRNA panel in a cohort of tumors that include cases that have undergone EMT transition validated by IHC (E-cadherin—negative). Moreover, we validated that dysregulation of these tumor suppressors and oncomiRs is an early event in lung cancer tumorigenesis that can be used as a diagnostic biomarker in cases of uncertainty. 

In addition, we performed separate analyses based on the histologic subtype for the 4 miRNAs. Hsa-miR-29b-3p, hsa-miR-205-5p, and hsa-miR-25-3p expression patterns were maintained without reaching statistical significance due to the low sample size. The expression of hsa-miR-181a-5p was significantly downregulated in tumor tissue for all three main histological subtypes, suggesting that hsa-miR-181a-5p silencing is an important, early, and consistent event in all types of lung cancer that is necessary for tumor initiation and progression. This pattern of alteration supports hsa-miR-181a-5p importance as both an early-stage biomarker and treatment target [41].

We further assessed the correlation between the IHC profile and miRNA expression. The differences in E-cadherin IHC were not associated with differences in miRNA expression. p53 IHC was associated with hsa-miR-29b-3p and hsa-miR-181a-5p expression. These two tumor suppressor miRNAs were upregulated in p53 IHC-positive lung cancer when compared with p53-negative tumors. This is a novel finding, especially considering that these two miRNAs are generally downregulated in tumor tissue [43,87]. We hypothesize that the upregulation of these two miRNAs in the context of pP53 mutation is due to a compensatory tumor suppressor role. A more in-depth study is necessary on this specific subtype of early-stage lung cancer to assess its outcome and therapy response. 

## 4. Materials and Methods

### 4.1. Patient Information

A cohort of 51 patients diagnosed between January 2013 and December 2016 with early-stage lung cancer and who was first-line surgically treated according to standard protocols were selected from the “Leon Daniello” Pneumology Institute database. This study was approved by the Ethics Committees of the “Leon Daniello” Pneumology Institute and The “Iuliu Hatieganu” University of Medicine and Pharmacy Cluj-Napoca. 

### 4.2. Baseline Data Collection

The demographic data collected were sex, age at diagnosis, and survival data. Outcomes of interest were overall survival (OS). OS was defined as the time from diagnosis to death. Survival status was censored at the latest follow-up date.

### 4.3. Morphological Characterization

Surgical samples were processed according to standard sampling protocols. Sections of interest were sampled, formalin-fixed, and paraffin-embedded. Hematoxylin and eosin (HE) staining was done on 3-µm-thick sections [88]. Histological diagnosis and staging were done on HE sections aided by classic diagnostic IHC for lung cancer based on the WHO Classification of Tumors of the Lung, Pleura, Thymus, and Heart [89]. Parameters analyzed for tumor and TME characterization include histology type, nuclear atypia, intratumor necrosis, stromal and intratumor inflammatory infiltrate, and the presence of tertiary lymphoid structures (TLS). 

### 4.4. Immunohistochemistry

IHC staining was performed using a fully automated slide preparation system (Benchmark GX, Ventana/Roche, Arizona, USA). IHC staining was used for E-cadherin, p53, CD4, and CD8 (Ventana/Roche antibodies). E-cadherin staining was scored according to intensity (0–no staining, 1–weak, 2–moderate, and 3–strong). Abnormal p53 expression was defined as moderate to strong nuclear positivity in more than 20% of tumor cells or complete loss of nuclear positivity [90]. TILs populations were assessed using the CD4 and CD8 staining. CD4 and CD8 positivity was calculated separately for the stromal and intratumor compartments, being assessed by the percentage of positive cells. According to the percentage of positive cells, five intensity levels were proposed: 0–5% absent, 5–15% low intensity, 15–25% moderate intensity, 25–50% high intensity, >50% very high intensity. The HE and IHC slides were individually assessed by two experienced pathologists. Where discordance was found, the cases were assessed in a panel and consensus was reached. 

### 4.5. Bioinformatics Analysis

To better understand the regulatory network between lung cancer and TME, we used the miRNet 2.0 online platform in which we introduced the 4 selected IHC markers studied together with two inflammatory cytokines, IL-10 and IL-6, that are commonly expressed in lung cancer TME [91]. Based on the network analysis and literature search, we selected 4 miRNAs that target *TP53* and regulate EMT in lung cancer (hsa-miR-25-3p, hsa-miR-29b-3p, hsa-miR-181a-5p, and hsa-miR-205-5p). 

Our selection includes two tumor suppressor (hsa-miR-29b-3p and hsa-miR-181a-5p) and two tumor promoter (hsa-miR-25-3p and hsa-miR-205-5p) miRNAs. This 4-miRNA panel will allow the assessment of the p53 pathway and EMT process in early-stage lung cancer tumorigenesis.

A heatmap for the 4 miRNAs was generated to evaluate their expression in various cancer and metabolic processes [92]. The impact of the 4 miRNAs on lung cancer patients’ survival analysis was done based on the TCGA database using the StarBase v2.0 online tool [93] (Figure 5). 

### 4.6. RNA Extraction

Tumor zones, percentage of tumor cells in the selected area, and adjacent peritumoral normal tissue were noted on a classic HE-stained slide. Tumor cellularity was noted on a 5 level percentual scale: 0–5%, 5–15%, 15–25%, 25–50%, >50%. Only tumor zones with more than 15% of tumor cells were marked for further isolation for molecular analysis. Tumor zones and adjacent normal tissue zones were selected by two experienced pathologists. Non-stained 10 μm slides were overlapped over the marked HE slide, and interest zones were isolated using a scalpel in two distinct collection tubes (tumor tissue and adjacent normal tissue). Total RNA was extracted from FFPE tissue using the Qiagen RNeasy FFPE Kit (Cat. No. 73504) based on the instructions furnished by the manufacturer’s protocol. The RNA concentration was measured with a NanoDrop-1000 spectrophotometer (Thermo Scientific, Waltham, MA, USA). 

### 4.7. cDNA and qRT-PCR

The cDNA synthesis was done using 50 ng of total RNA extracted from FFPE tissue, reverse-transcribed, and amplified using the TaqMan miRNA Reverse Kit (Applied Biosystems, Thermo Fisher Scientific) with primers/probes specific for each miRNA. The cDNA was then diluted and used for PCR. For the amplification, we used TaqMan Fast Advanced Master Mix (Applied Biosystems) and TaqMan miRNA assays: RNU48 (Cat. No. 001006); U6 (Cat. No. 001973); hsa-miR-29b-3p (Cat. No. 000413); hsa-miR-205-5p (Cat. No. 000509); hsa-miR-181a-5p (Cat. No. 000480); hsa-miR-25-3p (Cat. No 000403). The qRT-PCR data analysis was done using the ΔΔCt method, as previously described by Berindan-Neagoe et al. [94]. 

### 4.8. Statistical Analysis

Statistical analysis was performed with Graphpad Prism software version 8.0 (GraphPad, San Diego, CA, USA). The *t*-test was used to compare the differential expressions of miRNAs between tumor tissue and adjacent normal tissue. To compare differences between the morphological, immunohistochemical characteristics and tumor microenvironment cellular populations of major histological subtypes of lung cancers, the chi-squared test and Fisher’s exact test were used. Kaplan–Meier curves and log-rank tests were used to analyze OS. *P*-values lower than 0.05 were considered statistically significant.

## 5. Conclusions

We present an in-depth characterization of the TME in a cohort of 51 early-stage lung cancer patients. Our analysis showed that these tumors are immunologic-active tumors that present a moderate-to-high inflammatory TME in 80.4% of the cases and that the inflammatory infiltrate is localized in the stromal compartment, where the TLS were also found in 74.5% of the cases. The main component of the TME is represented by CD4 cells, which are the most abundant cellular population. The abundance of CD4 cells was associated with a higher expression of hsa-miR-181a-5p in the adjacent normal tissue. The intratumor compartment is scarce in TILs, and a minor increase in CD8 cells is seen at this level. 

In this study, we investigated a 4-miRNA panel consisting of two tumor suppressors and two oncomiRs that were altered in early-stage lung cancer tumors compared with the adjacent normal tissue. Among them, has-miR-181a-5p was found consistently downregulated in all lung cancer tumor types. Tumor suppressor miRhashsa-miR-29b-3p, down-regulated in advanced lung cancer, was upregulated in our early-stage lung cancer cohort, suggesting that its silencing occurs as a later event. A novel finding is represented by the upregulation of tumor suppressor miRNAs hsa-miR-29b-3p and hsa-miR-181a-5p in the p53 IHC-positive tumors.

The current diagnostic and therapeutic approaches in lung cancer care face major limitations that prevent early detection and effective treatment of these tumors. Therefore, an integrated approach to this heterogeneous pathology, including histology and molecular pathology, can allow us to assemble different pieces of the same puzzle in the quest for novel biomarkers and therapeutic target identification. 

## Figures and Tables

**Table 1:** Morphological, histological, and immunohistochemical characteristics of the 51 lung cancer patients.

Parameter	LUAD	LUSC	NE LC	Total	*p*-Value
	(*n* = 22)	(*n* = 21)	(*n* = 8)		
**Sex**					0.0615
F	6 (27.3%)	2 (9.5%)	4 (50%)	12 (23.5%)	
M	16 (72.7%)	19 (90.5%)	4 (50%)	39 (76.5%)	
**5-year survival**					0.7485
Deceased	8 (36.4%)	6 (28.6%)	2 (25%)	16 (31.4%)	
Alive	14 (63.6%)	15 (71.4%)	6 (75%)	35 (68.6%)	
**Pathological stage**					0.9821
IA	11 (50%)	9 (42.9%	4 (50%)	24 (47%)	
IB	8 (36.4%)	8 (38.1%)	3 (37.5%)	19 (37.3%)	
IIA	3 (13.6%)	4 (19%)	1 (12.5%)	8 (15.7%)	
**pT stage**					0.9831
pT1a	6 (27.3%)	6 (28.6%)	3 (37.5%)	15 (29.4%)	
pT1b	5 (22.7%)	3 (14.3%)	1 (12.5%)	9 (17.6%)	
pT2a	8 (36.4%)	8 (38.1%)	3 (37.5%)	19 (37.3%)	
pT2b	3 (13.6%)	4 (19%)	1 (12.5%)	8 (15.7%)	
**Differentiation grade**					*<0.0001* ****
Well-differentiated (G1)	4 (18.2%)	0 (0%)	5 (62.5%)	9 (17.6%)	
Moderately differentiated (G2)	9 (40.9%)	10 (47.6%)	0 (0%)	19 (37.3%)	
Poorly differentiated (G3)	9 (40.9%)	11 (52.4%)	1 (12.5%)	21 (41.2%)	
Undifferentiated (G4)	0 (0%)	0 (0%)	2 (25%)	2 (3.9%)	
**Intratumor necrosis**					*0.003* **
Absent	8 (36.4%)	0 (0%)	4 (50%)	12 (36.4%)	
Present	14 (63.6%)	21 (100%)	4 (50%)	39 (63.6%)	
**Nuclear Atypia**					*0.026* *
Absent	15 (68.2%)	8 (38.1%)	7 (87.5%)	30 (58.8%)	
Present	7 (31.8%)	13 (61.9%)	1 (12.5%)	21 (41.2%)	
**Stromal TILs**					*<0.0001* ****
Low	2 (9.1%)	1 (4.8%)	7 (87.5%)	10 (19.6%)	
Moderate	12 (54.5%)	10 (47.6%)	1 (12.5%)	23 (45.1%)	
High	8 (36.4%)	10 (47.6%)	0 (0%)	18 (35.3%)	
**Intratumor TILs**					*0.021* *
Absent	0 (0%)	0 (0%)	2 (25%)	2 (3.9%)	
Low	19 (86.4%)	17 (81%)	5 (62.5%)	41 (80.4%)	
Moderate	3 (13.6%)	4 (19%)	1 (12.5%)	8 (15.7%)	
**Tertiary lymphoid structures**					0.2146
Absent	5 (22.7%)	4 (19%)	4 (50%)	13 (25.5%)	
Present	17 (77.3%)	17 (81%)	4 (50%)	38 (74.5%)	
**Active germinative centers**					0.3231
Absent	9 (52.9%)	13 (76.5%)	3 (75%)	25 (65.8%)	
Present	8 (47.1%)	4 (23.5%)	1 (25%)	13 (34.2%)	
**Stromal CD4 TILs**					*<0.0001* ****
Low	0 (0%)	0 (0%)	4 (50%)	4 (7.8%)	
Moderate	0 (0%)	2 (9.5%)	2 (25%)	4 (7.8%)	
High	14 (63.6%)	13 (61.9%)	1 (12.5%)	28 (54.9%)	
Very High	8 (36.4%)	6 (28.6%)	1 (12.5%)	15 (29.5%)	
**Intratumor CD4 TILs**					0.1011
Absent	12 (54.5%)	8 (38.1%)	7 (87.5%)	27 (53%)	
Low	8 (36.4%)	11 (52.4%)	1 (12.5%)	20 (39.2%)	
Moderate	0 (0%)	2 (9.5%)	0 (0%)	2 (3.9%)	
High	2 (9.1%)	0 (0%)	0 (0%)	2 (3.9%)	
**Stromal CD8**					0.0615
Absent	0 (0%)	1 (4.8%)	2 (25%)	3 (5.9%)	
Low	7 (31.8%)	6 (28.6%)	3 (37.5%)	16 (31.4%)	
Moderate	9 (40.9%)	5 (23.8%)	3 (37.5%)	17 (33.3%)	
High	6 (27.3%)	9 (42.8%)	0 (0%)	15 (29.4%)	
**Intratumor CD8 TILs**					0.2261
Absent	12 (54.5%)	6 (28.6%)	6 (75%)	24 (47%)	
Low	7 (31.8%)	12 (57.1%)	1 (12.5%)	20 (39.2%)	
Moderate	2 (9.1%)	3 (14.3%)	1 (12.5%)	6 (11.8%)	
High	1 (4.5%)	0 (0%)	0 (0%)	1 (2%)	
**E-cadherin**					0.6692
Moderate intensity (2+)	14 (63.6%)	12 (57.1%)	6 (75%)	32 (62.7%)	
High Intensity (3+)	8 (36.4%)	9 (42.9%)	2 (25%)	19 (37.3%)	
**p53**					0.1401
Absent	9 (40.9%)	5 (23.8%)	5 (62.5%)	19 (37.3%)	
Positive	13 (59.1%)	16 (76.2%)	3 (37.5%)	32 (62.7%)	

LUSC—lung squamous cell carcinoma. LUAD—lung adenocarcinoma. NE LC—neuroendocrine lung cancer. TILs—tumor-infiltrating lymphocytes; * *p* ≤ 0.05; ** *p* ≤ 0.01; **** *p* ≤ 0.0001.

**Table 2:** Expression pattern and modulatory roles of the 4 selected miRNAs.

miRNA	Regulation Status TCGA	Roles in Cancer	Ref.
hsa-miR-25-3p	↑ LUSC, ↑ LUAD	OncomiR, EMT activation through PTEN & FOXP2.	[36,37]
hsa-miR-29b-3p	↑ LUAD	Tumor suppressor miRNA, EMT pathway, chemotherapy, and radiotherapy resistance.	[38,39,40]
hsa-miR-181a-5p	↓ LUSC, ↓ LUAD	Tumor suppressor miRNA, EMT pathway, angiogenesis, proliferation.	[41,42,43]
hsa-miR-205-5p	↑ LUSC, ↑ LUAD	OncomiR, TP53IN1 targeting, EMT pathway, proliferation, metastasis,	[44,45]

## Data Availability

The data underlying this article cannot be shared publicly to maintain the privacy of individuals that participated in the study. The data will be shared upon reasonable request to the corresponding author.
